# Effectiveness of Applying Hyperbranched PVAc Copolymer Emulsion for Ecological Sand-Fixing in the High Salt-Affected Sandy Land

**DOI:** 10.3390/polym17172403

**Published:** 2025-09-03

**Authors:** Meilan Li, Yayi Jin, Jiale Wan, Wei Gong, Keying Sun, Liangliang Chang

**Affiliations:** 1 Key Laboratory of Comprehensive Utilization of Tailings Resources in Shaanxi Province, Shangluo University, Shangluo 726099, China; liecho2009@163.com (M.L.); 15596378600@163.com (Y.J.); wjl18791795062@163.com (J.W.); s18329640080@sina.com (K.S.); 231026@slxy.edu.cn (L.C.); 2 Chengdu Institute of Organic Chemistry, Chinese Academy of Sciences, Chengdu 610041, China

**Keywords:** salty desert, hyper-branched sand-fixing emulsion, inorganic salts, sand fixation effect, ecological restoration

## Abstract

This research seeks to reduce wind-blown sand hazards in saline deserts by introducing hyperbranched PVAc copolymer emulsion as a novel ecological sand-fixing material. The study began with the preparation of the emulsion, then evaluated its fundamental properties and the salt tolerance of latex films through FTIR, SEM, and mechanical strength assessments. The sand-fixing properties (compressive strength, anti-water erosion, anti-wind erosion, thermal aging, freeze–thaw stability, and water retention) were evaluated. In addition, their effects on increasing both the growth of microbes and plants in salty deserts have been evaluated by field experiments to understand their ecological effects. The experimental results showed that the hyperbranched PVAc copolymer emulsion has excellent salt resistance and can be used as an ecological sand-fixing material in salty deserts. The research findings demonstrate that the hyperbranched PVAc copolymer emulsion exhibits superior salt tolerance, rendering it an effective ecological sand-fixing material for saline deserts. Notable attributes encompass its capacity to significantly mitigate NaCl-induced aggregate damage to sand-fixing materials, thereby enhancing sand fixation performance; its robust thermal aging resistance, freeze–thaw stability, and salt tolerance, which enable it to withstand environmental temperature variations; and experimental assessments of sand-based plant and microbial growth confirming favorable ecological impacts. This study presents novel methodologies for designing ecological sand-fixing materials in saline deserts to combat desertification.

## 1. Introduction

The combined effects of marginal water irrigation and traditional agricultural practices have accelerated global soil degradation, manifesting as salinization, nutrient depletion, and desertification [1,2,3,4]. These interconnected processes pose a significant threat to agricultural sustainability and socioeconomic stability worldwide. Currently, over 100 countries globally are affected by desertisation in salt-affected soils, with approximately one billion hectares of salt-affected land concentrated primarily in arid and semi-arid regions across Asia, Africa, Australia, and South America [5,6]. In China, over 99.13 million hectares of salt-affected soil suffer desertisation [7]. Saline desertification usually generates multiple adverse environmental consequences, such as dust storms, damage to crops, spreading of polluted sediments, loss of fertilizer, and deposition of soil in ditches [8,9,10,11]. Therefore, the desertification of salty deserts is one of the most serious problems confronting sustainable agriculture in semi-arid and arid regions. The Horqin Salt-affected Sandy Land, situated in the agropastoral transition zone between the Inner Mongolian Plateau and Northeast Plains, has experienced accelerated desertification since the 1970s [12,13,14]. Intensive land-use pressures coupled with aeolian processes have triggered widespread soil salinization, creating an imperative for systematic ecological rehabilitation and sustainable comprehensive management [15].

To solve this problem, humans around the world have been forced to consider measures to reduce or prevent desertisation. Currently, sand fixation technologies can be broadly classified as mechanical, biological, chemical, and comprehensive sand fixation [16,17,18,19]. Generally, saline soil reclamation has been carried out using halophytic vegetation, such as *Sesuvium portulacastrum* L., *Clerodendron inerme Gaertn.*, *Suaeda maritima Dum.*, *Ipomoea pescaprae Sweet*, *Heliotropium curassavicum* L., and one tree species *Excoecaria agallocha* L. [20]. However, due to severe wind erosion in highly saline sandy areas, vegetation restoration alone resulted in very low seed germination rates. Based on the above-mentioned problems found in the present study, stabilizing sand under high-salinity conditions is considered a critical factor in restoring salt-affected desert ecosystems.

Commonly used raw materials for sand fixation today include polymers, silicate minerals, and bio-based composites [21,22,23,24]. In general, polymer materials, as new sand-fixing materials to prevent sand-wind erosion, have received much attention [25,26,27]. These include polyacrylamide, polyvinyl alcohol, vinyl acetate-ethylene copolymer emulsion, and polyurethane [28,29,30]. For example, polyurethane (PU), developed by Japan’s TORAY, has been tested in China’s saline sandy areas of Qinghai. Initial results demonstrated strong sand-fixing effects shortly after application. However, over time, the material began to pulverize and rapidly lost its stabilizing capacity [31]. Additionally, the high cost of PU makes it impractical for large-scale use. Therefore, performance and cost became the main considerations for the materials applied in the sand-fixing of high-salt-affected sandy land.

Usually, emulsion polymers, particularly those based on vinyl acetate monomers, offer significant cost advantages while maintaining excellent adhesive properties [32,33,34,35]. Owing to their unique characteristics, these emulsions have emerged as a primary solution for combating desertification and soil salinization. Vinyl acetate-dibutyl maleate (VAc-DBM) copolymer emulsions have gained prominence because they combine essential performance characteristics with low production costs, making them widely applicable for these purposes [35]. By analyzing and studying the salt-tolerant behavior of polymer materials, it was considered that hyperbranched copolymers had the most appropriate salt-tolerant characteristics for future applications because they contained a high branching structure and a large number of terminal hydrophilic functional groups, such as hydroxyl and carboxyl groups, reducing the permeation of salt ions, due to their promising salt tolerance, these materials have attracted significant research attention in both academic and industrial settings [36]. As a result, a new eco-friendly sand-fixing polymer was developed specifically for saline desert restoration.

Therefore, the main purpose of this study was to evaluate the sand fixation ability of a P(VAc-DBM-AA-AM-IA-HBP) hyperbranched emulsion in a salty desert land. The properties of P(VAc-DBM-AA-AM-IA-HBP) were investigated through adsorption, contact angle, gas permeability, mechanical property, wind, thermal aging, freeze–thaw resistance, and water retention tests. Additionally, their effects on increasing both the growth of microbes and plants in salty deserts have been evaluated to understand their ecological effects. The results of this study are anticipated to produce an eco-friendly sand stabilization material for salty sand land, offering novel ideas for global desertification mitigation, while establishing both theoretical and practical support for degraded ecosystem restoration.

## 2. Experimental Section

### 2.1. Materials

Itaconic acid (IA) and triethanolamine (TEA) were purchased from Alfa Chemical Reagent Corporation (Zhengzhou, China). The vinyl acetate (VAc) and dibutyl maleate (DBM) employed in this study were obtained from Sichuan Weinilun Industry Corp. (Chongqing, China) and utilized as received, without additional purification. Succinic acid was purchased from Shanghai Hans Chemical Co., Ltd. (Shanghai, China). Acrylamide (AM) and Acyclic acid (AA) were purchased from Kelong Chemical Reagent Corporation (Chengdu, China). K_2_S_2_O_8_ and N, N-dimethylformamide were purchased from Sinopharm Chemical Reagent Co. (Beijing, China). The nonionic surfactant Pluronic L35 was obtained from Haian Petrochemical Corp. (Nantong, China) and employed as received. Deionized water was used in all the preparations.

### 2.2. Procedure of Preparing P(VAc-DBM-AA-AM-IA-HBP) Hyperbranched Emulsion

Synthesis of Hyperbranched Copolymer Monomer IA-HBP

**Figure 1:**
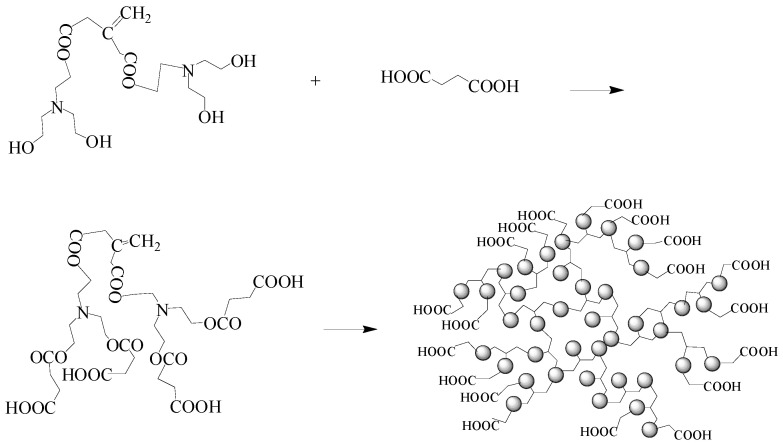
The preparation of IA-HBP.

**Figure 2:**
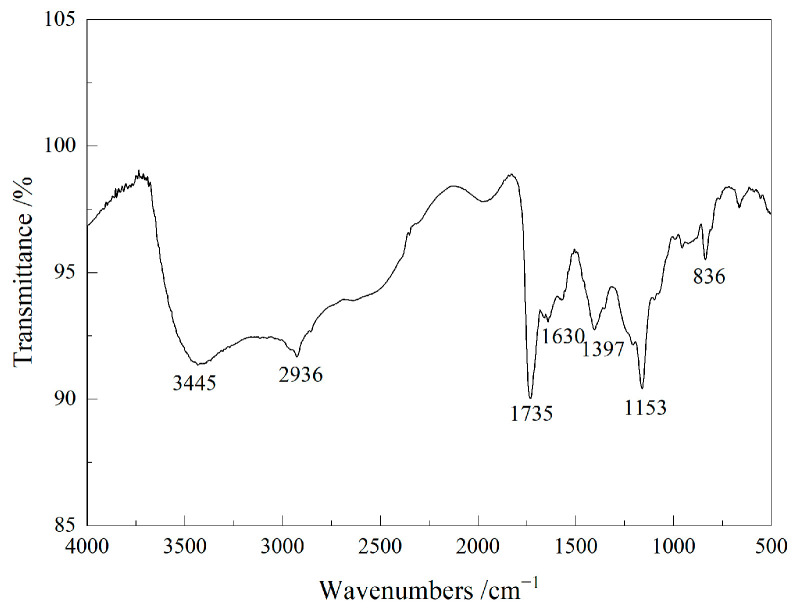
The FTIR spectrum of IA-HBP.

**Figure 3:**
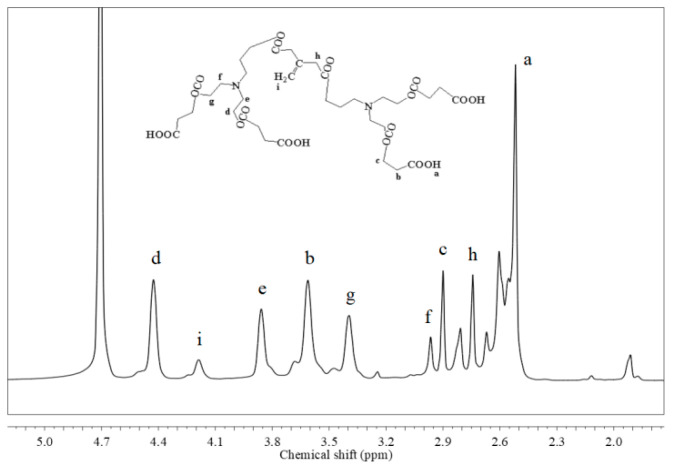
The ^1^H-NMR spectrum of IA-HBP.

The hyperbranched copolymer monomer, IA-HBP, was synthesized, as shown in Figure 1. 0.15 mol of itaconic acid and triethanolamine (29.84 g) were added to a 250 mL three neck flask which was equipped with a thermometer, mechanical stirrer, and reflux condenser. When 50 mL N, N-dimethylformamide was added to the flask, and the reaction process was started with a stirring speed of 240 rpm for 15 min to yield a completely dissolved reaction system, the temperature was raised to 125 °C and the mixture was stirred for 4 h. 0.08 mol Succinic acid was then added and the mixture was stirred at 135 °C for 4 h. The entire preparation was performed under a N_2_ atmosphere. Subsequently, the yellow samples were collected and concentrated using a rotary evaporator at 120 °C for 2 h and dried under reduced pressure to obtain IA-HBP. The Fourier transform infrared spec-trometer (Nicolet 6700, Thermo Fisher Scientific, Waltham, MA, USA) and ^1^H-NMR (Bruker Ascend 600, Bruker, Billerica, MA, USA) spectra of IA-HBP are shown in Figure 2 and Figure 3, respectively.

Preparation of the P(VAc-DBM-AA-AM-IA-HBP) hyperbranched emulsion

A four-necked flask (250 mL) was prepared with a stirring paddle, a condenser, three dropping funnels for monomers and initiator solution, and an air outlet to mix the 16.7 g emulsifier (SDS and L35), 0.15 g buffer (Na_2_CO_3_), and 120 mL distilled water under air atmosphere. The system was continuously heated to 63 °C with a stirring speed of 150 r/min and stirred for 30 min. The reaction temperature was elevated to 72 °C, at which point 30 wt% of the initiator (KPS) solution (10 g total mass) was introduced rapidly. Following a 30 min interval, simultaneous dropwise addition commenced of 20 wt% mixed oil-soluble monomers (50.2 g VAc, 15.73 g DBM, and 2.3 g AA) and 20% aqueous monomer solution (6.79 g AM, 11.33 g IA-HBP), maintained at 76 °C throughout the 30 min addition period for pre-emulsion preparation.

After termination of the circumfluence phenomenon, the residual monomer mixture and initiator solution were introduced dropwise into the four-neck flask over a period of at least 4 h. Subsequently, the emulsion pH was adjusted to neutrality using 1 M Na_2_CO_3_ solution. The reaction mixture was then maintained at 88 °C to ensure complete consumption of residual monomers and initiator, typically requiring approximately two hours for complete polymerization. Following this, the reactor contents were cooled to ambient temperature and filtered to remove residual coagulum, yielding the P(VAc-DBM-AA-AM-IA-HBP) hyperbranched emulsion. The synthesis experiment was verified through three independent trials, and the averageconversion rate of the P(VAc-DBM-AA-AM-IA-HBP) polymer was calculated to be 94.6%.

### 2.3. The Basic Properties of the P(VAc-DBM-AA-AM-IA-HBP) Hyperbranched Emulsion

10 mL P(VAc-DBM-AA-AM-IA-HBP) sand-fixing agent emulsion was pipetted into an EP tube and frozen in a refrigerator until complete solidification was achieved. The EP tube was then transferred to a freeze-dryer for a 3-day lyophilization process, during which the solid sample transformed into a porous foam structure. Finally, exactly 20 mg of the dried foam sample was weighed and analyzed using a Fourier Transform Infrared (FT-IR) spectrometer.

The key emulsion properties influencing sand stabilization efficacy encompass viscosity, average particle diameter, particle size distribution, and ζ-potential. These parameters were quantified using an LVDV-C viscometer (Brookfield Engineering Laboratories, Middleboro, MA, USA), dynamic light scattering (DLS), and laser Doppler micro-electrophoresis performed with a Zetasizer Nano ZS instrument (Malvern Instruments Ltd., Malvern, UK).

### 2.4. The Salt-Tolerance Properties of Latex Film

All emulsions were prepared and cast into membranes under ambient conditions, resulting in membranes of uniform size and thickness. Salt solutions with concentrations ranging from 0.0 to 5.0 wt% were prepared using deionized water, in which the membranes were subsequently immersed for 72 h at room temperature. The water absorption characteristics under varying salinity conditions were determined through gravimetric analysis.

### 2.5. Sand-Fixing Properties of the Emulsion

#### 2.5.1. Adsorption Test

The adsorption kinetics of the emulsion on sand were determined using batch adsorption tests [37]. The emulsion (20 g) was weighed into a 100 mL beaker, and then sand doses of 60 g were added and deposited for 0, 15, 30, 45, 60, 80, and 100 min and 2, 3, 6, 9, 12, and 24 h. An NP-30S vertical centrifuge was utilized for the experimental procedure. Beakers underwent immediate centrifugation at 4000 rpm for 30 min. Subsequently, the supernatant was collected into separate containers. The equilibrium adsorption of emulsion onto sand, qe (mg/g), was then calculated using the mass balance equation Equation (1).

q_e_ = (C_0_ − C_e_) V/m (1)

where C_0_ and C_e_ (mg/L) represent the initial and equilibrium concentrations of the curing agent, respectively; V (L) denotes the volume of the solution; and m (g) indicates the mass of sand.

The adsorption kinetics of the emulsion onto sand were investigated using two established kinetic models: the pseudo-first-order and pseudo-second-order models.

#### 2.5.2. Preparation of Fixed Sand Specimen

Standardized specimens were prepared by precisely weighing 10 g of sand, 0.3 g of salt, and 1 g of 2.0% (*w*/*w*) emulsion, followed by homogeneous mixing using a mechanical stirring device. The mixture was then transferred into cylindrical stainless steel molds (2.0 cm diameter × 2.2 cm height) and compacted with a static compression device under 0.5 MPa pressure for 30 s to form structurally consolidated sand columns. The resulting specimens were cured under constant temperature and humidity conditions (25 ± 0.5 °C, RH 50 ± 5%) for 4 h, followed by 7-day drying under identical conditions, after which uniaxial compressive strength testing was conducted using a universal testing machine at a loading rate of 1 mm/min.

#### 2.5.3. Compressive Resistance of Stabilized Sand Samples

The compressive strength was determined using a universal testing machine (WDW-5; Jinan Chuanbai Instrument Co., Jinan, China). The fully cured sand columns were subjected to uniaxial compression at a constant displacement rate of 100 mm/min until failure. The peak stress was recorded as the compressive strength (MPa), and triplicate measurements were performed for each sample group to ensure statistical reliability.

#### 2.5.4. Investigation of Water Retention Characteristics

Within the laboratory, a cylindrical sand column (height: 35–45 mm, diameter: 90 mm) was established to assess the water retention capacity of sand-fixing materials. A 90 mm diameter container held a homogeneous mixture of 100 g sand and 3 g salt. Following the application of emulsions at varying concentrations to form a surface crust, water was applied to the sand surface at a rate of 1 L/m^2^. The moist specimens were subsequently dried in an oven maintained at 25 °C. The moisture content of each saline sand sample was then determined at hourly intervals over a 24 h period (mean of three replicates), calculated based on weight loss measurements:

Water content = [(wet sand weight − dry sand weight)/water weight] × 100%

#### 2.5.5. Experimental Investigation of Water Erosion Resistance

A laboratory apparatus was developed to evaluate the erosion resistance of sand columns against water. Specifically, 100 g of sand was homogeneously mixed with 3 g of salt and placed within a 90 mm diameter container. Following the application of a precisely controlled emulsion spray, a consolidated crust layer formed on the surface. Moist samples were subsequently dried to constant mass in an oven maintained at 60 °C. A calibrated uniform water distributor then simulated rainfall at an intensity of 10 L·m^−2^ to assess the erosion resistance of the sand-fixing material. The mass loss of the sand-fixing system was recorded at 1 min intervals over a 25 min duration. Measurements represent the mean of three replicate readings per time point, with the entire test procedure repeated in triplicate.

#### 2.5.6. Anti-Wind Erosion Experiment

An anti-wind erosion apparatus was designed and constructed to evaluate the wind erosion resistance of sand columns. For the experimental procedure, a mixture comprising 100 g sand, 3 g salt, and 10 g emulsion (3.0% concentration) was prepared and subsequently dried at 60 °C for 12 h. Wind speeds of 12, 14, 16, 18, and 20 m/s were simulated. The mass loss of the sand-fixing system was recorded at 5 min intervals over a 40 min duration (mean value derived from triplicate measurements). The entire experiment was performed in three independent replicates.

#### 2.5.7. Thermal Aging Ability of Fixed Sand Specimen

The specimen column was subjected to heat aging in an air circulation oven at 60 °C for a continuous period of 10 days, with a cycle. The compressive strength of each sample was measured after each cycle to evaluate the thermal aging stability of the sand-fixing material.

#### 2.5.8. Freezing-Thawing Resistance Tests

Freezing-thawing tests were conducted from −20 °C to 60 °C based on the climatic features of the desert. Freeze–thaw tests were conducted using automatic freeze–thaw equipment (TL-010, Suzhou Zhihe Environmental Instrument Co., Ltd., Suzhou, China). One cycle of the freeze–thaw process involved 22 h of freezing at −20 °C and 2 h of thawing at 60 °C. The specimen column was subjected to 28 such cycles, and then subjected to a compressive strength test to obtain its freeze–thaw stability related to the sand-fixing material used. The cited results are the average of three replicates.

### 2.6. Ecological Effect of the Sand-Fixing Emulsion

#### 2.6.1. Plants Growth

A simulated ecological sand stabilization experiment was conducted in the Horqin saline sandy region, involving the seeding of *Festuca arundinacea* (Tall Fescue), a halophytic species, into saline sandy soil. An emulsion was subsequently spray-applied to the soil surface for sand fixation, followed by monitoring of halophyte establishment and development.

#### 2.6.2. Microorganism Analysis

Four months later, triplicate soil samples (0–20 cm depth) were collected from each saline-affected sandy site for microbial analysis. Microbial populations in the salt-impacted sandy soils were quantified using standard plate count methodology.

#### 2.6.3. Effects of Sand-Stabilizing Amendments on the Physico-Chemical Properties of Sandy Soils

The physicochemical properties of salinity-affected sandy soil treatments were assessed by employing standardized analytical protocols: organic matter content was quantified via the potassium dichromate volumetric method; pH was measured using a glass electrode; and total nitrogen was determined by the Kjeldahl procedure. Available phosphorus and potassium concentrations were analyzed using inductively coupled plasma atomic emission spectrometry (ICP-AES).

## 3. Results and Discussion

In practical applications, optimal ecological sand-fixing materials necessitate chemical stability, environmental safety, and climatic adaptability alongside maintaining cost-effectiveness. These materials should exhibit high performance, durability, and economic viability. Following these fundamentals, we developed a novel P(VAc-DBM-AA-AM-IA-HBP) hyperbranched emulsion for application in high-salt-affected sandy lands.

### 3.1. Analysis of FTIR Spectroscopy

**Figure 4:**
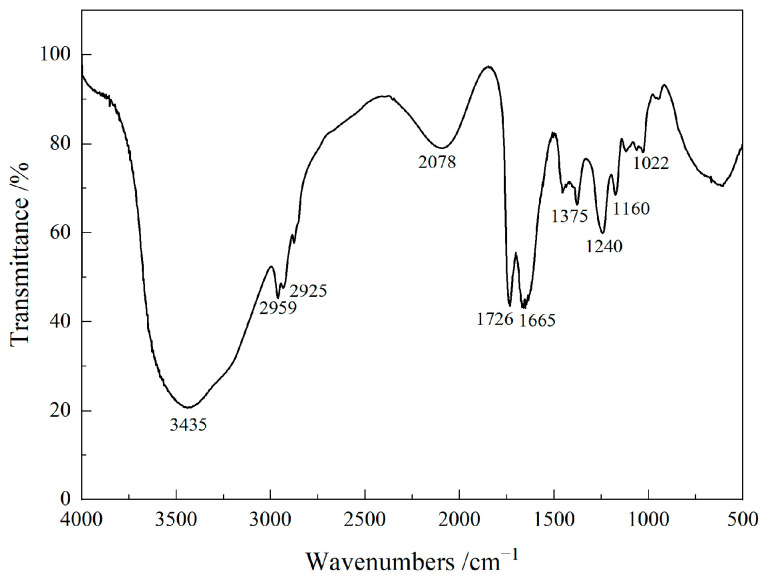
The FTIR spectrum of P(VAc-DBM-AA-AM-IA-HBP) emulsion.

The chemical structure of the P(VAc-DBM-AA-AM-IA-HBP) hyperbranched emulsion was characterized using a Nicolet 170SX FTIR spectrometer (Thermo Nicolet Co., Madison, WI, USA) across the spectral range of 500–4000 cm^−1^. Figure 4 presents the FTIR spectra for this emulsion. Distinct absorption peaks at 3435 cm^−1^ and 1726 cm^−1^ correspond to O-H and C=O stretching vibrations in carboxylic acids, respectively, indicating substantial carboxyl functional groups on the copolymer surface. Asymmetric stretching vibrations of C-O-C were detected near 1240 cm^−1^, while symmetric stretching vibrations appeared at approximately 1022 cm^−1^. Moreover, the absorption band observed at approximately 2936 cm^−1^ was assigned to the C-H stretching vibrations of methyl and methylene groups. Concurrently, the C-N stretching vibration at 1160 cm^−1^, along with characteristic amide absorption bands at 1665 cm^−1^, provides evidence for copolymerization between TEA and IA. Furthermore, the C=C stretching vibration absorption at 1630 cm^−1^ disappeared in the FTIR spectrum of the P(VAc-DBM-AA-AM-IA-HBP) copolymer. These results showed that copolymerization occurred between VAc, DBM and AA, AM, and IA-HBP, as expected, and the functional groups remained unaffected during polymerization. Based on these findings and corroborating the literature [38], the P(VAc-DBM-AA-AM-IA-HBP) copolymer was successfully synthesized.

### 3.2. Fundamental Characteristics of the P(VAc-DBM-AA-AM-IA-HBP) Emulsion

The fundamental characteristics of emulsions, including particle size, viscosity, and zeta potential, exert a direct influence on sand fixation efficacy in practical applications. Consequently, investigation into emulsion properties is paramount to practical utilization. The characteristics of the P(VAc-DBM-AA-AM-IA-HBP) emulsions are presented in Table 1. As shown in Table 1, the emulsion particles exhibited diameters below 1 mm, demonstrating effective penetration capability into aeolian sand pores with subsequent crust formation. Furthermore, the low polydispersity index (PDI < 0.35) confirmed successful polymerization, resulting in monodisperse particle distribution. The P(VAc-DBM-AA-AM-IA-HBP) emulsion had a higher viscosity than P(VAc-DBM-AA-AM) once IA-HBP was integrated into the emulsion structure. The main reason for this phenomenon is that IA-HBP containing high-density polar groups (-COOH) can induce interparticle crosslinking or form a spatial network [39]. During this phase, the specific surface area of the emulsion particles decreased, while their surface charge density increased. The interaction forces between the emulsion particles and the solvent intensified, accompanied by amplified entanglement effects among molecular chains within the emulsion. This heightened resistance to particle mobility necessitated the application of greater force during shearing of the P(VAc-DBM-AA-AM-IA-HBP) emulsion system. Consequently, the viscosity of the emulsion increased. The analytical results indicated that the measured viscosity was optimal for sand fixation applications in saline desert environments.

### 3.3. Salt Tolerance Property of the P(VAc-DBM-AA-AM-IA-HBP) Latex Film

**Figure 5:**
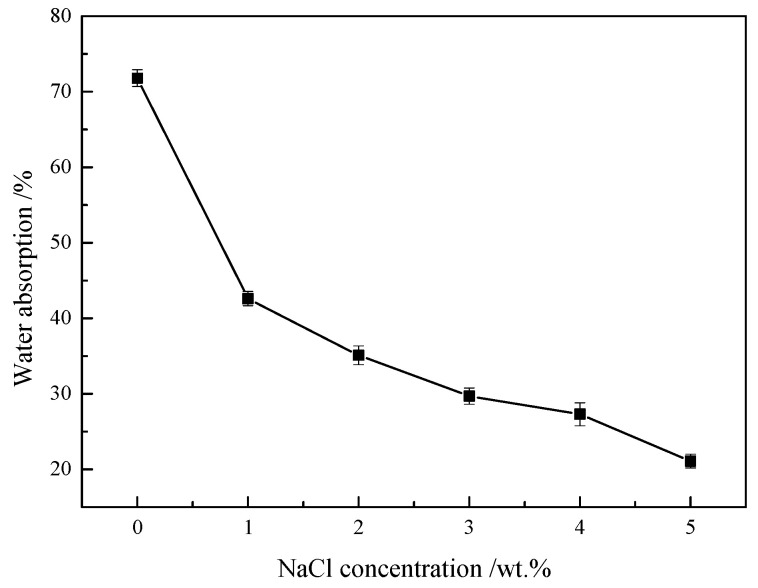
The Dependency of Latex Film Water Absorption on NaCl Concentration in Aqueous Medium.

It is well established that intensive evaporation promotes salt accumulation in topsoil layers and elevates salinity levels, which can significantly compromise the sand fixation efficacy of polymeric emulsions. To ensure the optimal performance of latex films for dune stabilization under varying saline conditions, a systematic evaluation of their water absorption characteristics under different NaCl concentrations is imperative. The influence of the ionic strength on the water absorption of the latex film was evaluated by changing the ionic strength of the NaCl solution, as shown in Figure 5. The experimental results revealed a distinct two-stage absorption behavior: a precipitous decline in water absorption occurred as NaCl concentration increased from 0.0 wt% to 1.0 wt%, followed by a progressively moderated reduction trend when the concentration further rose from 1.0 wt% to 5.0 wt%. There are three main reasons for this phenomenon: First, the three-dimensional hyperbranched topology of IA-HBP generated spatial confinement effects, effectively blocking salt ion diffusion pathways through polymeric matrices owing to its densely packed architecture, thereby improving the salt rejection performance. Second, with increasing NaCl concentration, the elevated osmotic pressure (Δπ ≈ 2cRT) of the solution diminishes the water absorption driving force [40]. Concurrently, the charge screening effect of Na^+^ on ionized polymer groups (-COO^−^) compressed the electric double-layer structure and attenuated electrostatic repulsion forces, thereby inducing contraction of the polymer network. Finally, Cl^−^ competitively disrupted the hydration shells surrounding polar polymer groups (e.g., -COOH), which increased the ionic strength and consequently enhanced the hydrophobic effect, ultimately leading to further tightening of the molecular chain conformations. Consequently, the water absorption of the P(VAc-DBM-AA-AM-IA-HBP) latex film decreased. These results showed that the latex film had excellent salt tolerance and could meet the requirements of sand fixing under varying NaCl concentrations in high-salt-affected sandy land areas.

### 3.4. Influence of Salt Concentration Levels on the Mechanical Characteristics of Membranes

**Figure 6:**
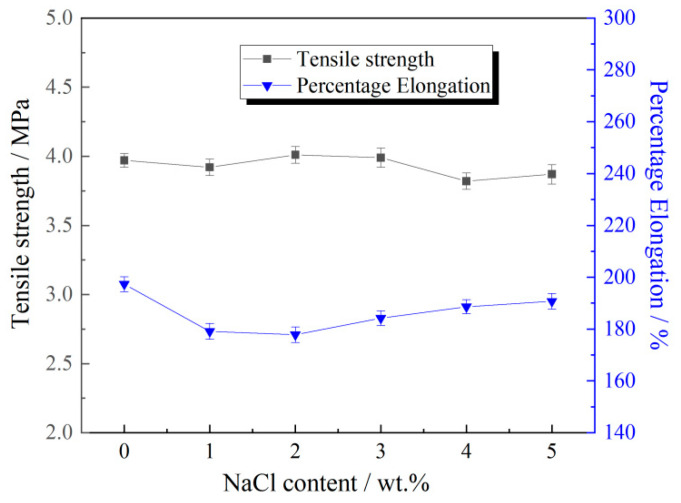
Effects of Sodium Chloride Concentration on Tensile Strength and Elongation Percentage of Polymer Films.

It is well established that intensive evaporation induces salt accumulation in topsoil layers and elevated salinity levels, which significantly affects the sand fixation efficacy of polymeric emulsions. To ensure the latex film satisfies the sand-fixation requirements in saline desert environments, it is necessary to assess its mechanical properties across varying NaCl concentrations. As illustrated in Figure 6, the effects of ionic strength were systematically evaluated through controlled NaCl solution variations. The experimental data demonstrated remarkable stability in tensile strength (3.93 ± 0.1 MPa after 720 h immersion) independent of NaCl concentration (0–5%). These findings confirm that the P(VAc-DBM-AA-AM-IA-HBP) latex film possesses exceptional salt tolerance. This confirms that P(VAc-DBM-AA-AM-IA-HBP) is technically suitable for sand stabilization projects in hypersaline desert environments.

### 3.5. Morphological Features of Membranes Across Varying Salinity Levels

**Figure 7:**
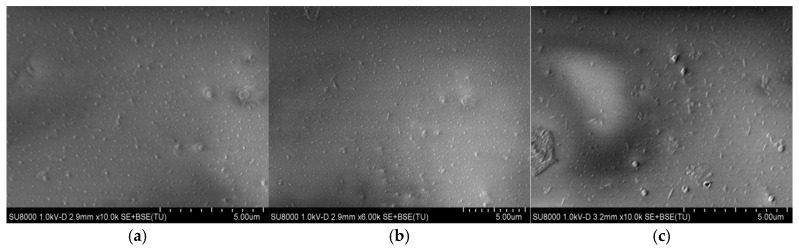
Micrographs of Film Surface Morphology under Different Salt Concentrations: (**a**) 2.0 wt.% NaCl; (**b**) 3.0 wt.% NaCl; (**c**) 4.0 wt.% NaCl.

To investigate the influence of salt concentration on the structural characteristics of latex films, scanning electron microscopy (SEM) was utilized to characterize the morphological features of membrane surfaces under controlled saline conditions. Latex films were immersed in NaCl solutions of varying concentrations for 720 h and then dried at 50 °C. As shown in Figure 7, with increasing salt concentration, NaCl crystals formed only on the surface of the latex film, without penetrating the polymer molecular chains or damaging the film structure. Since the salt content in the saline sandy land was generally below 3%, the results indicated that the structure of the latex film remained unaffected by the NaCl concentration of the saline deserts, demonstrating excellent salt resistance. This confirmed that the P(VAc-DBM-AA-AM-IA-HBP) latex film can be effectively applied as a sand-fixing material for saline desert remediation.

### 3.6. Hydrophilic Performance of P(VAc-DBM-AA-AM-IA-HBP) Latex Film

**Figure 8:**
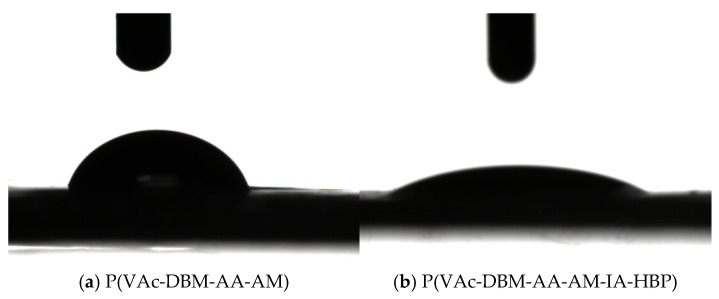
Contact angle of P(VAc-DBM-AA-AM) (**a**) and P(VAc-DBM-AA-AM-IA-HBP) (**b**) latex films.

Water contact angle measurements and corresponding optical images for the latex films are presented in Figure 8a,b. Latex films were considered hydrophilic if their water contact angles were less than 90 degrees. As shown in Figure 8, both the latex films exhibited hydrophilic properties. Compared to P(VAc-DBM-AA-AM) latex film, the P(VAc-DBM-AA-AM-IA-HBP) latex film demonstrated a smaller water contact angle, measuring 22.61°, indicating that the surface of the P(VAc-DBM-AA-AM-IA-HBP) latex film exhibited stronger hydrophilicity compared to the P(VAc-DBM-AA-AM) latex film. The main reason for this phenomenon is that the three-dimensional branched structure in IA-HBP induces an expansion of the intermolecular chain spacing, which reduces the interaction forces between emulsion molecular chains. Simultaneously, the incorporation of numerous hydrophilic groups (e.g., –OH and–COOH) facilitated the active dispersion of these hydrophilic groups on the exterior of the molecular chains [41]. Consequently, the P(VAc-DBM-AA-AM-IA-HBP) latex films exhibited excellent hydrophilicity.

### 3.7. The Gas Permeability of P(VAc-DBM-AA-AM-IA-HBP) Latex Film

**Figure 9:**
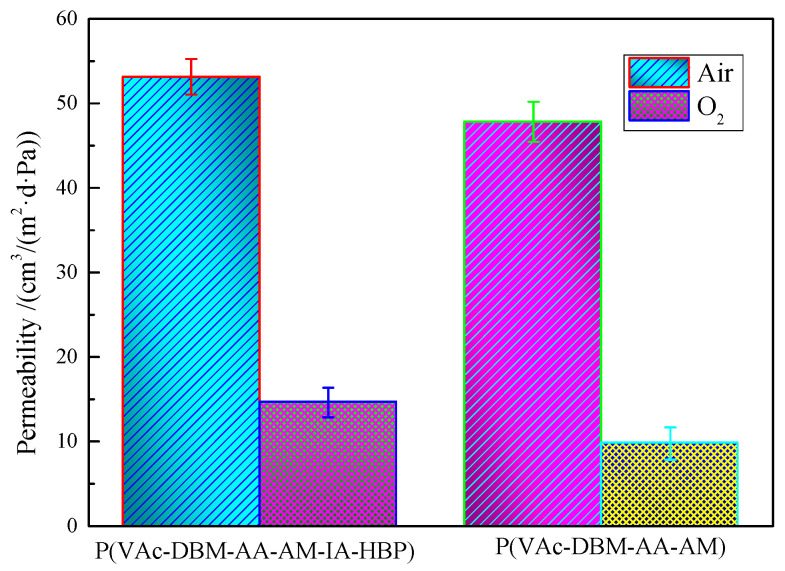
The gas permeability of P(VAc-DBM-AA-AM-IA-HBP) latex film.

As illustrated in Figure 9, the air permeability of the P(VAc-DBM-AA-AM-IA-HBP) latex film significantly surpassed that of the P(VAc-DBM-AA-AM) latex film. This enhancement can be attributed to the three-dimensional branched architecture and abundant free-volume voids inherent in the IA-HBP molecules. Consequently, it is evident that all latex films synthesized in this investigation exhibit measurable permeability characteristics and fail to provide effective air isolation. Furthermore, Figure 9 demonstrates that the oxygen transmission rate (OTR) for the P(VAc-DBM-AA-AM-IA-HBP) latex film paralleled the permeability characteristics observed for air. Hence, it may be anticipated that oxygen, as well as other atmospheric gases including CO_2_ and N_2_ can effectively permeate through the P(VAc-DBM-AA-AM-IA-HBP) latex film. Moreover, P(VAc-DBM-AA-AM) latex film and P(VAc-DBM-AA-AM-IA-HBP) latex film exhibited OTR values of 9.75 cm^3^/(m^2^·d·Pa) and 14.61 cm^3^/(m^2^·d·Pa) respectively, with P(VAc-DBM-AA-AM-IA-HBP) displaying the higher OTR among the two samples. These results confirm that the incorporation of IA-HBP enhanced the OTR of the P(VAc-DBM-AA-AM-IA-HBP) latex film, which could be attributed to the distinctive molecular structural characteristics of IA-HBP. First, large interchain spacing and abundant free-volume cavities were created by the 3D branched structure, forming continuous gas transport channels. Second, the lower crystallinity and reduced intermolecular forces improve the chain segment mobility [42]. Finally, the steric hindrance effect of the terminal functional groups (e.g., -OH and -COOH) further prevented tight molecular chain packing.

### 3.8. Basic Sand-Fixing Performance

#### 3.8.1. Adsorption Analyses

**Figure 10:**
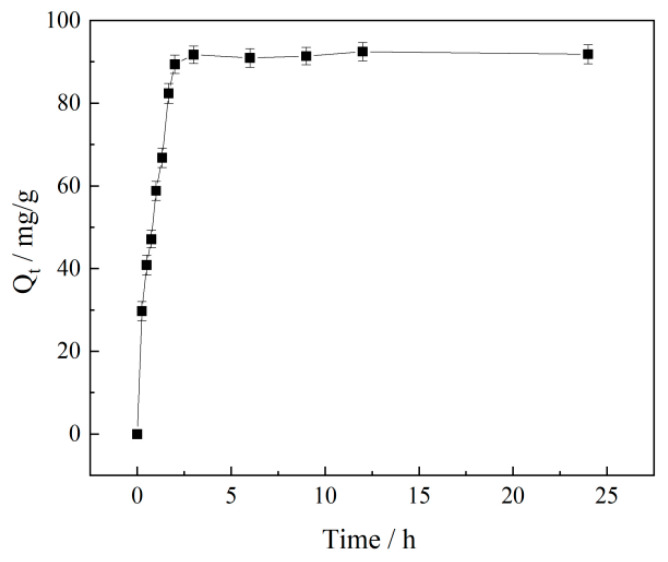
Effects of the adsorption time on sand adsorption by the P(VAc-DBM-AA-AM-IA-HBP) emulsion.

Figure 10 demonstrates that the adsorption capacity of the P(VAc-DBM-AA-AM-IA-HBP) emulsion onto sand exhibited a rapid increase during the initial two hours of the experiment. This change in adsorption may be attributed to the rapid distribution of the abundant P(VAc-DBM-AA-AM-IA-HBP) emulsion functional hydrophilic groups on the sand surface, leading to the rapid accumulation of the adsorbents. At the 2 h adsorption time point, the P(VAc-DBM-AA-AM-IA-HBP) emulsion adsorption capacities on the sand reached 89.41 mg/g. After adsorption for 2 h, the amount of P(VAc-DBM-AA-AM-IA-HBP) emulsion adsorbed on the sand did not change significantly, and the maximum adsorption amount remained at approximately 90 mg/g. The contact time required to reach the adsorption equilibrium was 2 h.

**Figure 11:**
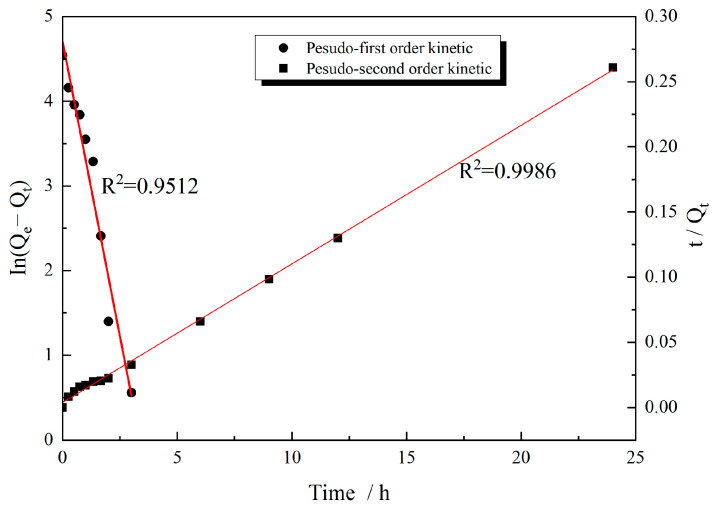
Experimental Results and Kinetic Model Fitting for P(VAc-DBM-AA-AM-IA-HBP) Emulsion Adsorption on Sand.

To further evaluate the main adsorption mechanism, we analyzed the obtained adsorption data for the adsorption of the P(VAc-DBM-AA-AM-IA-HBP) emulsion on the sand by using both pseudo-first-order and pseudo-second-order kinetic models [43] (Figure 11); the adsorption parameters are shown in Table 2. Figure 11 shows that the pseudo-second-order model can better describe the adsorption behavior of the P(VAc-DBM-AA-AM-IA-HBP) emulsion on sand. This conclusion was supported by the higher correlation coefficient (R^2^ = 0.9986) and closer agreement between the calculated and experimental equilibrium adsorption capacities. This finding indicates that sand adsorption onto the P(VAc-DBM-AA-AM-IA-HBP) emulsion occurred primarily via physicochemical complexation rather than mechanical interlocking, with chemical adsorption significantly strengthening the emulsion-sand interaction.

#### 3.8.2. Water-Resistant Performance of P(VAc-DBM-AA-AM-IA-HBP) Consolidated Layers

**Figure 12:**
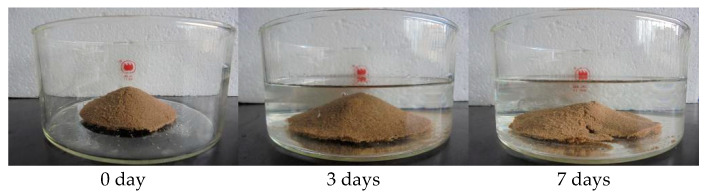
The disintegration performance of treated sand specimens in water.

In high-salinity sandy areas, environmental factors could affect the sand-fixing effectiveness of the copolymer emulsion film, especially in desert regions with such natural rainfall; therefore, the sand-fixing agent’s sand-fixing effectiveness improves when its consolidation layer withstands water-resistant performance for a longer period. Figure 12 illustrates the disintegration resistance of P(VAc-DBM-AA-AM-IA-HBP) consolidated layers. Following 72 h of aqueous immersion, the specimens maintained structural integrity relative to unimmersed controls, exhibiting minimal observable sand detachment from their surfaces. After 7 days of soaking, it was observed that the consolidated layers developed minor cracks in water but exhibited no significant structural changes. This indicates that the copolymer emulsion could effectively maintain the aggregation of sand under rainwater erosion.

**Figure 13:**
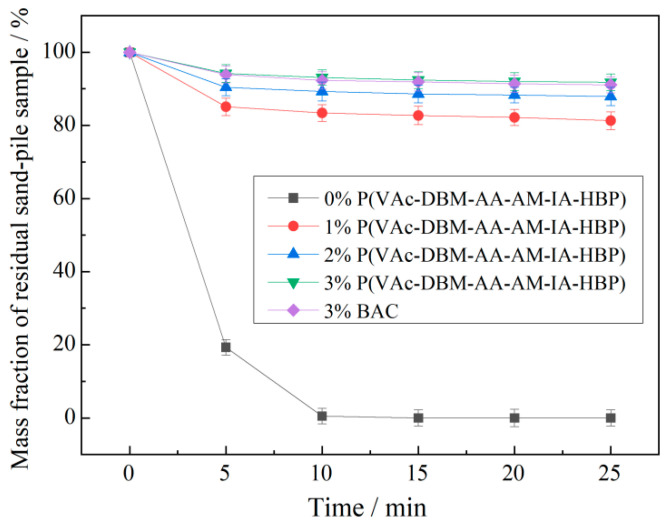
P(VAc-DBM-AA-AM-IA-HBP)’s anti-water erosion ability.

As illustrated in Figure 13, the resistance of P(VAc-DBM-AA-AM-IA-HBP) to water erosion exhibited a positive correlation with increasing concentration. At a 3% concentration, the water retention characteristics of sandy soil amended with P(VAc-DBM-AA-AM-IA-HBP) were comparable to those of sandy soil treated with an equivalent concentration of bio-based attapulgite copolymer (BAC) [44], demonstrating the polymer’s robust resistance to water erosion. This enhancement arises from the three-dimensional hyperbranched architecture, which generates significant steric hindrance, coupled with the presence of multiple branching sites that form a physical network analogous to chemical crosslinks. This structural configuration ensures the material’s integrity during water exposure. Therefore, sand mixed with the P(VAc-DBM-AA-AM-IA-HBP) emulsion held water and prevented sandy soil erosion by water.

#### 3.8.3. Compressive Strength of P(VAc-DBM-AA-AM-IA-HBP) Consolidated Layers

**Figure 14:**
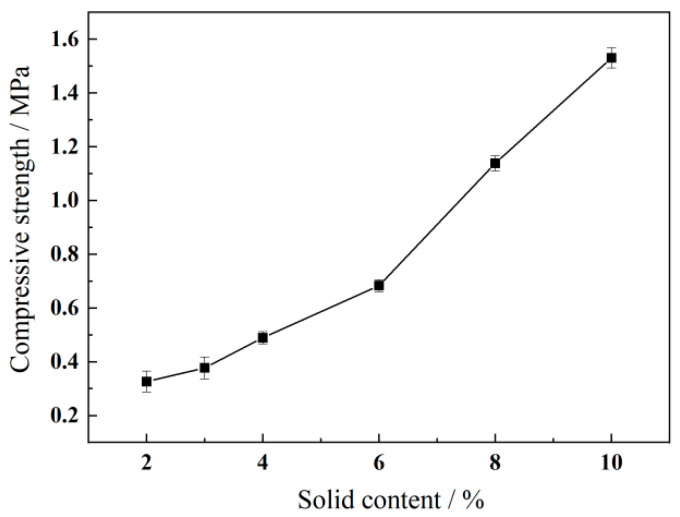
Evaluation of Compressive Strength in Emulsions at Varying Solid Contents.

Compressive strength, representing the mechanical resistance of the sand-fixing crust, serves as an indicator of interparticle bonding effectiveness. The experimental sand, collected from mobile dunes in Qinghai Sandy Land, naturally contains approximately 3% salt content due to proximity to saline lakes. Following the methodology outlined in Section 2.5.2, we prepared test specimens by mixing this saline sand with emulsion concentrations ranging from 2.0% to 10.0%. The compressive strength of each fixed sand specimen is shown in Figure 14; the results demonstrated a positive correlation between emulsion concentration and compressive strength. This relationship stems from enhanced interparticle bonding density, where increased emulsion content facilitates more complete sand particle coverage and stronger interfacial interactions, thereby improving mechanical resistance. In addition, the specimens fixed by the P(VAc-DBM-AA-AM-IA-HBP) emulsion with 3% concentration exhibited compressive strengths of 0.377 MPa, meeting the required compressive strength. This indicates that even low concentrations of P(VAc-DBM-AA-AM-IA-HBP) can provide the desired mechanical strength to resist larger external forces without cracks. Furthermore, comparative field tests against the polyurethane (PU) formulation developed by TORAY of Japan revealed that the compressive strength of sand surfaces treated with the P(VAc-DBM-AA-AM-IA-HBP) emulsion remained at 0.341 MPa after 30 days of application. These results collectively demonstrate that the P(VAc-DBM-AA-AM-IA-HBP) emulsion constitutes a superior candidate material for soil stabilization in high-salinity sandy environments.

#### 3.8.4. SEM Analysis

**Figure 15:**
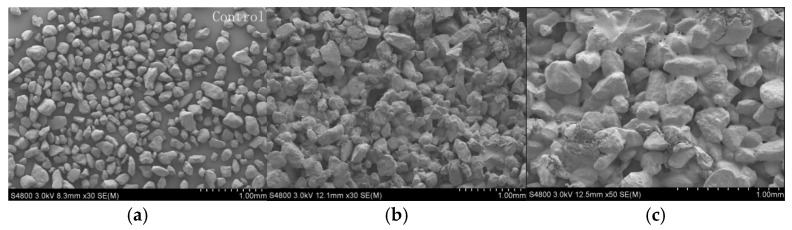
Surface morphology images of sand fixed with various emulsions: (**a**) unfixed; (**b**) fixed with 3.0% P(VAc-DBM-AA-AM); (**c**) fixed with 3.0% P(VAc-DBM-AA-AM-IA-HBP).

To investigate how sand-fixing materials influence sand fixation performance, scanning electron microscopy (SEM) was utilized to perform a detailed surface microstructure analysis on sand treated with various emulsions. As shown in Figure 15, the untreated specimens exhibited discontinuous transition zones surrounding individual sand particles, characterized by open pore networks and limited particle-to-particle (Figure 15a); therefore, the weak interparticle cohesion facilitated wind erosion, significantly increasing susceptibility to dust emission events.

Nonetheless, sand treated with 3.0% P(VAc-DBM-AA-AM) and P(VAc-DBM-AA-AM-IA-HBP) exhibited dense interfacial contacts within the inter-particle transition zone, characterized by frequent overlapping or intergranular bonding between adjacent sand grains. The polymeric long-chain molecules enveloped particle surfaces, establishing intermolecular bonds and forming an interconnected network that produced a viscoelastic crust at the sand interface (Figure 15b,c). The formation of a surface crust significantly improved interparticle cohesion, thereby enhancing the overall stability of the sand matrix. Moreover, because the P(VAc-DBM-AA-AM-IA-HBP) emulsion had better hydrophilic performance, when the emulsion was mixed with the soil, more P(VAc-DBM-AA-AM-IA-HBP) emulsion could be adsorbed on the surface of the soil particles and gradually formed a more compact adhesive layer.

#### 3.8.5. Anti-Wind Erosion Performance of P(VAc-DBM-AA-AM-IA-HBP) Consolidated Layers

**Figure 16:**
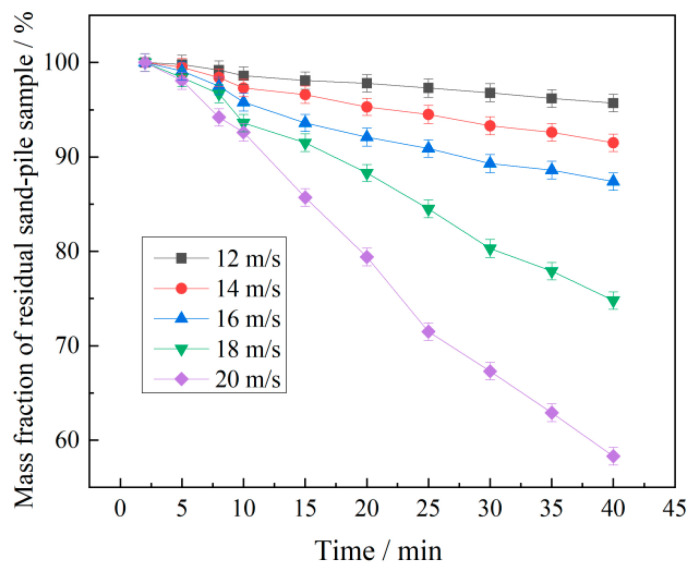
Anti-wind erosion performance of the sand-pile models with different wind velocities.

Figure 16 presents the results of wind erosion resistance tests conducted via physical simulations employing natural wind exposure on sand pile models. The erosion rate exhibited a consistent increase with escalating wind velocity. The polymer P(VAc-DBM-AA-AM-IA-HBP) achieved sand fixation rates exceeding 70% at wind velocities of 12, 14, 16, and 18 m/s. Even at 20 m/s, it maintained a 55% sand-fixing efficacy, satisfying wind erosion resistance criteria for desert environments [45]. This phenomenon can be attributed to the hyperbranched carboxyl groups within P(VAc-DBM-AA-AM-IA-HBP) functioning as robust ionic moieties. Their potent ionization capacity expands the hydrodynamic volume of macromolecular chains, while the bulky pendant groups enhance molecular chain rigidity. Consequently, the introduction of inorganic electrolytes does not induce a significant reduction in macromolecular coil dimensions. Significantly, the hyperbranched architecture also reduces interfacial tension between sand particles and the emulsion, thereby improving wettability and interfacial bonding strength.

#### 3.8.6. Thermal Aging Ability of P(VAc-DBM-AA-AM-IA-HBP) Consolidated Layers

**Figure 17:**
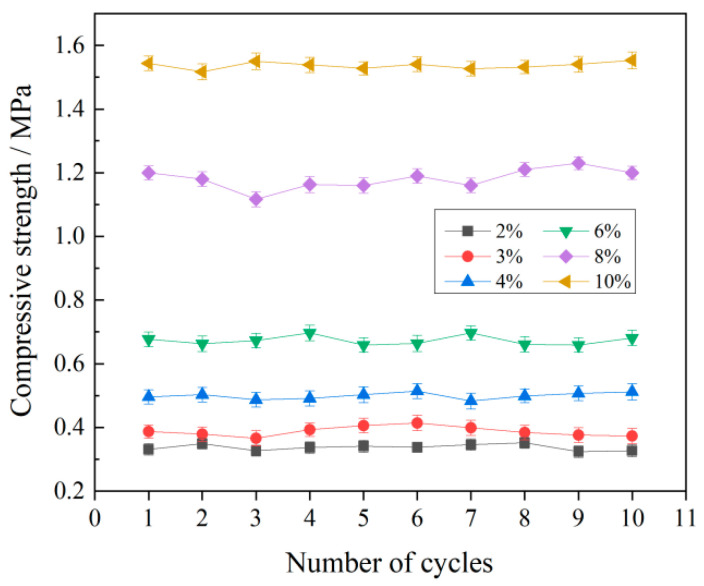
Variations in Compressive Strength of Stabilized Sand Specimens with Thermal Cycling Frequency.

To investigate the influence of temperature on the sand-stabilizing efficacy of the emulsion, sand containing 3% NaCl, treated with varying emulsion concentrations, was subjected to continuous thermal aging experiments. Figure 17 illustrates the correlation between compressive strength and the number of thermal aging cycles. The results indicate that compressive strength exhibited no significant variation across all samples throughout the 10-cycle testing period, regardless of increasing thermal aging cycles. This observation demonstrates the robust thermal aging resistance inherent in the P(VAc-DBM-AA-AM-IA-HBP) consolidated sand layers.

#### 3.8.7. Freeze–Thaw Ability of P(VAc-DBM-AA-AM-IA-HBP) Consolidated Layers

**Figure 18:**
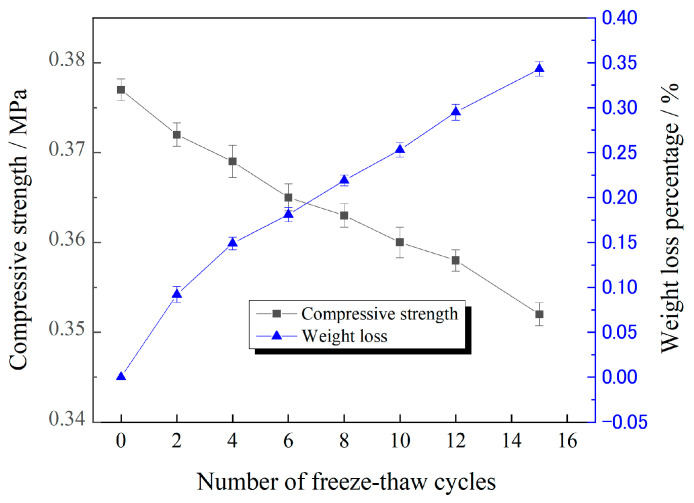
Variation Characteristics in Compressive Strength and Weight Loss of Test Specimens as a Function of Freeze–Thaw Cycle Frequency.

To assess the freeze–thaw stability of P(VAc-DBM-AA-AM-IA-HBP) consolidated layers, sand containing 3% NaCl was treated with a 3.0% (*w/w*) emulsion. The treated specimens were subjected to compressive strength testing and measurement of mass loss percentage. Figure 18 demonstrates a progressive decline in compressive strength across all samples with increasing freeze–thaw cycles. Nevertheless, after 28 cycles, the sample maintained a compressive strength of 0.345 MPa, still satisfying the requisite standard. The mass loss percentage reached 0.501% following 28 cycles. Although minimal, this mass loss contributed to the diminished compressive strength observed in the consolidated layers. This degradation primarily stems from spalling of the surface layer, which induces structural deterioration within the pore system of the hardened material [46]. These findings indicate that P(VAc-DBM-AA-AM-IA-HBP) consolidated layers exhibit good frost resistance and are suitable for sand fixation in saline desert environments.

#### 3.8.8. Water-Retaining Property of P(VAc-DBM-AA-AM-IA-HBP) Emulsion

**Figure 19:**
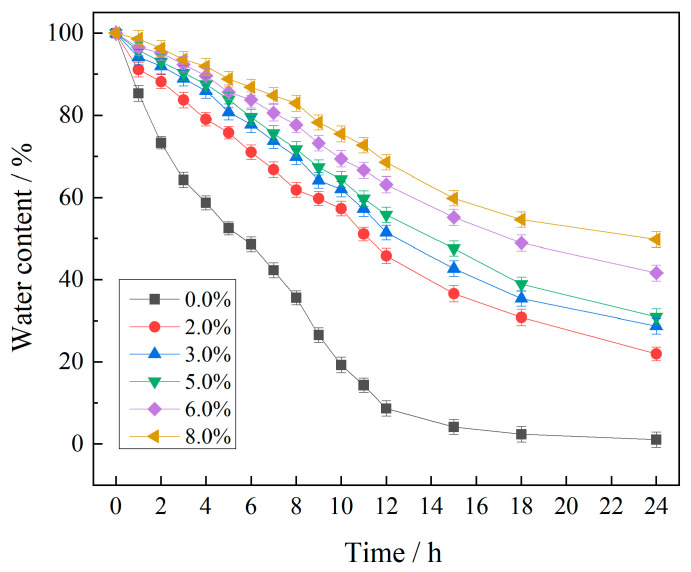
Water retention performance of P (VAc-DBM-AA-AM-IA-HBP) copolymer emulsion.

One of the key factors affecting seed germination and plant growth in high-salinity sandy soils is surface water availability. Therefore, the water retention properties of the P(VAc-DBM-AA-AM-IA-HBP) emulsion are highly significant for the ecological restoration of salty deserts, as shown in Figure 19. Throughout the experimental period, the moisture content in treated samples consistently exhibited significantly higher values compared to untreated samples. This implies that the P(VAc-DBM-AA-AM-IA-HBP) emulsion significantly enhances the water retention capacity of the sand samples. Moreover, the water retention effect was positively correlated with the dosage of P(VAc-DBM-AA-AM-IA-HBP). This improvement can be attributed to two primary mechanisms. First, the strong adhesive properties of the copolymer emulsion effectively spliced loose soil particles together, forming a dense and continuous layer on the sand surface [47]. This structural modification transformed the originally coarse water-conducting channels between sand particles into finer capillary channels, significantly reducing the water evaporation rates and effectively inhibiting moisture loss. Second, the IA-HBP component contains numerous strongly anionic and highly water-soluble carboxyl groups (-COOH) at its terminals, which easily convert free water into bound water, thereby further enhancing the water retention effect. Additionally, as the concentration of the copolymer emulsion increased, the sand surface film exhibited fewer defects, leading to improved water retention performance. In arid sandy lands, Pujol et al. [48,49] observed that increased water availability was the primary factor affecting seed germination. In other words, the use of the P(VAc-DBM-AA-AM-IA-HBP) copolymer emulsion may promote the germination and growth of psammophytes, making it highly beneficial for ecological restoration in high-salinity sandy soils.

Experimental assessments of thermal aging, freeze–thaw resistance, and water retention demonstrate that the P(VAc-DBM-AA-AM-IA-HBP) copolymer emulsion exhibits resilience to salt desert climate fluctuations and functions effectively in sand stabilization applications within high-salinity sandy soils.

### 3.9. Ecological Effect of P(VAc-DBM-AA-AM-IA-HBP) Copolymer Emulsion

#### 3.9.1. Modifications of Plant Growth Induced by P(VAc-DBM-AA-AM-IA-HBP) Copolymer Emulsion

**Figure 20:**
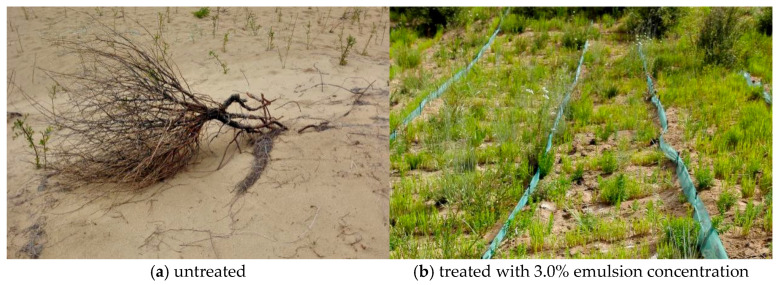
Parallel images of growth of Tall Fescue.

Figure 20 illustrates the development of Tall Fescue on saline sand treated with the P(VAc-DBM-AA-AM-IA-HBP) copolymer emulsion across a three-month span from April to June. In this period, we found an apparent disparity in the growth of Tall Fescue on sand-fixed and unfixed salty sand. Figure 20a shows the growth of Tall Fescue on unfixed salty sand, and Figure 20b shows the growth of Tall Fescue in salty sand fixed by the P(VAc-DBM-AA-AM-IA-HBP) copolymer emulsion. The Tall Fescue thrived in the fixed salty sand with P(VAc-DBM-AA-AM-IA-HBP) copolymer emulsion, and compared to the untreated soil samples, the Tall Fescue seed survival rate in soil treated with P(VAc-DBM-AA-AM-IA-HBP) copolymer emulsion can exceed 70%. Moreover, stem biomass, and plant height were all significantly higher than those in the control group. The luxuriant growth of the plants implied the effectiveness of sand-fixing materials in high-salinity sand-land restoration [50]. Emulsion forms interparticle bridges between mobile sand dunes, binding together sand particles to create an erosion-resistant crust. This solidified layer establishes a stable and favorable microenvironment conducive to vegetative colonization. And the molecular structure of P(VAc-DBM-AA-AM-IA-HBP) copolymer emulsion, particularly highlighting the presence of hydrolyzable ester bonds (from VAc and DBM) and hydrophilic functional groups (from AA, AM, and IA), which suggests, with relevant literature [51], polymers with such structures can be susceptible to microbial degradation and hydrolysis under environmental conditions, although the process may be slower in arid desert soils. This indicates that the P(VAc-DBM-AA-AM-IA-HBP) copolymer emulsion is an environmentally friendly sand-fixing agent. However, the primary objective persists in achieving sustained vegetation persistence and flourishing within stabilized saline sandy soil ecosystems. Further research is needed and should be related to both materials and Tall Fescue.

#### 3.9.2. Changes in Physicochemical Properties Caused by P(VAc-DBM-AA-AM-IA-HBP) Copolymer Emulsion

The basic physicochemical properties of saline sandy soil treated with P(VAc-DBM-AA-AM-IA-HBP) copolymer emulsion are shown in Table 3. Compared with the unfixed salty sand, the organic matter content, total nitrogen, available phosphorus, and available potassium levels of sandy soil with the copolymer emulsion increased significantly, indicating that the P(VAc-DBM-AA-AM-IA-HBP) copolymer emulsion could increase the content of nutrient elements in soil, which was beneficial to plants and microorganisms. Moreover, experimental results demonstrate that following fixation, the mean particle size of sand increased significantly. This phenomenon thereby reduced wind erosion rate due to enhanced surface roughness induced by coarser sand particles. Notably, larger sand particle sizes promote surface coarsening, mitigate erosion intensity, and elevate concentrations of organic carbon and total nitrogen within sandy soil.

#### 3.9.3. Changes in Sand Microecology Caused by the P(VAc-DBM-AA-AM-IA-HBP) Copolymer Emulsion

Table 4 demonstrates that microbial biomass in the saline sandy soil exhibited robust growth during the entire experimental duration. As shown in Table 4, in desert ecosystems characterized by extreme environmental stressors, actinomycetes consistently dominate microbial communities, exhibiting significantly higher abundance than bacteria and fungi. Compared with the control group, fixation of P(VAc-DBM-AA-AM-IA-HBP) markedly enhanced the average growth rate of beneficial soil microorganisms, which efficiently degrade soil organic pollutants and contribute to the restoration of soil ecological integrity. The rapid increase induced by P(VAc-DBM-AA-AM-IA-HBP) can be attributed to the enhanced sand surface roughness and water retention, which reduced wind erosion and fostered plant germination, ultimately improving microbe-friendly conditions for microbial growth. The observed increase in microbial populations within the sandy land may be largely attributed to the enhanced decomposition of plant residues, which elevates soil organic matter content and subsequently promotes plant growth. However, the inhibitory effect of P(VAc-DBM-AA-AM-IA-HBP) fixation on fungal populations remains unclear, warranting further investigation to facilitate the broader application of these sand-fixing materials.

## 4. Conclusions

Because desertified saline soils are a significant global concern, a hyperbranched PVAc copolymer emulsion was designed as an ecological sand stabilizer for salt-affected desert regions. The study revealed that even a small amount of emulsion could achieve optimal sand-fixing performance, including high compressive strength to mitigate wind erosion, as well as superior thermal aging and freeze–thaw resistance to endure extreme temperature fluctuations in saline deserts. Furthermore, water retention tests confirmed that the emulsion-treated sand maintained superior water retention and minimized evaporation. Combined with its exceptional salt resistance, it is highly suitable for sand fixation in salt-rich desert environments. The emulsion can increase soil fertility in terms of soil organic matter content, total nitrogen, available phosphorus, and available potassium content, as well as soil agglomerate structure. Sufficient hydrophilicity and O_2_ permeability of the emulsion can also promote the growth of plant and soil microbes in a better environment. Notably, biological assays further demonstrated that the treated sand had positive effects on both plant growth and microbial activity, indicating its ecological compatibility. These findings demonstrate that the P(VAc-DBM-AA-AM-IA-HBP) emulsion holds great promise as an eco-friendly sand-fixing material, particularly for the ecological restoration of highly saline sandy environments.

## Figures and Tables

**Table 1:** The fundamental property parameters for the P(VAc-DBM-AA-AM-IA-HBP) emulsion.

Emulsion Sample	Particle Size (nm)	Particle Size Distribution	Viscosity (mPa s)	Zeta Potential (mV)
P(VAc-DBM-AA-AM-IA-HBP)	317.6	0.31	194.7	−38.54
P(VAc-DBM-AA-AM)	201.72	0.26	103.6	−33.46

**Table 2:** Kinetic data for the adsorption of P(VAc-DBM-AA-AM-IA-HBP) on sand.

	Pseudo-First-Order Kinetic Model			Pseudo-Second-Order Kinetic Model		
Q_e_ (exp)/mg/g	K_1/_h^−1^	Q_e_/mg/g	R^2^	K_2_/g/mg/h	Q_e_/mg/g	R^2^
89.41	1.3919	110.255	0.95123	0.0293	93.985	0.9986

**Table 3:** Differences in physicochemical properties of soil samples before and after treatment with the emulsion.

Sample	Organic Matter Content /g/kg	Total Nitrogen /g/kg	Available Phosphorus /mg/kg	Available Potassium /mg/kg	Particle Size Composition/%		
					>0.425 mm	0.425~0.25 mm	<0.25 mm
Control	7.33	0.16	0.72	2.19	13.79	44.17	42.04
Emulsion	19.82	1.35	11.38	5.61	42.16	50.71	7.13

**Table 4:** Number of microbes in sand fixed with P(VAc-DBM-AA-AM-IA-HBP) four months later.

Sample	Bacteria/per g	Actinomycetes/per g	Fungi/per g
Control	3.11 × 10^6^	2.47 × 10^7^	1.82 × 10^5^
P(VAc-DBM-AA-AM-IA-HBP)	5.74 × 10^8^	1.05 × 10^9^	5.97 × 10^4^

## Data Availability

The data used to support the findings of this study are available from the corresponding author upon request due to privacy reasons.
